# A Study of Pigment, Adhesive, and Firing Temperature in Pottery Figurines Excavated from the Tomb of Qibi Ming, China

**DOI:** 10.3390/molecules28237739

**Published:** 2023-11-24

**Authors:** Yanli Li, Haiqiang Guo, Ke Xiao, Panpan Liu, Xiaolian Chao, Peng Fu, Huiping Xing, Yuhu Li

**Affiliations:** 1 Engineering Research Center of Historical Cultural Heritage Conservation, Ministry of Education, School of Materials Science and Engineering, Shaanxi Normal University, Xi’an 710119, China; lyl01211106@snnu.edu.cn (Y.L.); liupanpan@snnu.edu.cn (P.L.); chaoxl@snnu.edu.cn (X.C.); 2 Xianyang Museum, Xianyang 712039, China; guohaiqiang0304@163.com (H.G.); xiaoke@163.com (K.X.); 3 Shaanxi Institute for the Preservation of Cultural Heritage, Xi’an 710075, China

**Keywords:** painted pottery figurines, pigment, adhesives, micro-Raman spectroscopy, Py-GC/MS, DIL

## Abstract

Some painted pottery figurines were excavated from the tomb of Qibi Ming of the Tang Dynasty. A series of analytical techniques were employed to understand the craftsmanship of these painted pottery figurines. The pigment, cross-section, adhesive, and firing temperature were analyzed using microscopy (OM), energy X-ray fluorescence spectrometry (EDX), micro-Raman spectroscopy, pyrolysis–gas chromatography–mass spectrometry (Py-GC/MS), and a dilatometer (DIL). The results demonstrated that the surface of the pigment layers had degraded to different degrees. The pigment particles were litharge, gypsum, malachite, cinnabar, hematite, minium, white lead, and carbon black. The cross-sectional images show that the painted layer of figurines 10-0966 and 10-0678 included a pigment layer and a preparation layer. The preparation layer of both pigments was lead white. Animal glue was used as an adhesive. The firing temperature of the pottery figurines was likely 1080 °C. This study can provide more accurate information with regard to the composition of the raw materials utilized in the making of these artifacts and support the selection of appropriate substances for the purposes of conservation and restoration of the painted pottery figurines.

## 1. Introduction

**Figure 1:**
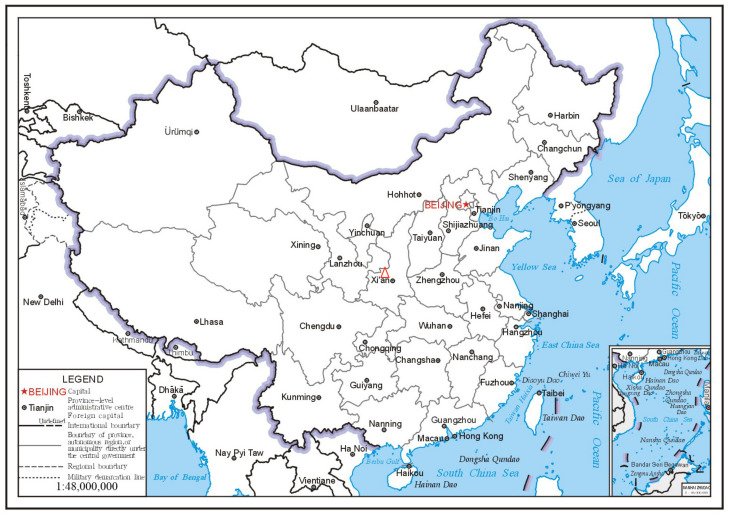
Locality map of the tomb of Qibi Ming.

A tomb dating back to the Tang Dynasty (618-907 AD) was excavated starting from 1973 by the Xianyang Museum and the Department of History of the Nanjing University, in China. The tomb is located in Xianyang, China (Figure 1). The tomb’s occupant was Qibi Ming. He was the son of Qibi Heli, a famous minority general in the early Tang Dynasty, who served as the general of Zuoying Yangwei and the governor Helan. Qibi Ming was born in the 23rd year of Zhenguan (649 AD) and died in the first year of Zhengsheng (695 AD) in the Guzang county, in Liangzhou. In the first year of Wansui Tiantong (696 AD), he was buried in the tomb in Xianyang. He was a brave warrior with outstanding achievements and made important contributions to the political stability of the Tang Dynasty [1,2].

There was a large amount of sealed soil on the ground of the tomb, which has been completely eroded. Only an irregular cone with a height of 4.8 m and a circumference of about 42 m remains in the western part of the front and rear chambers. The entire tomb consists of a tomb passage, a passage, a courtyard, a small niche, a front and rear corridor, and a front and rear tomb chamber. The length of the tomb is 65 m and its depth is 18.25 m. The tomb’s passage has a long, sloping shape and was filled with rammed earth during the burial [1].

**Figure 2:**
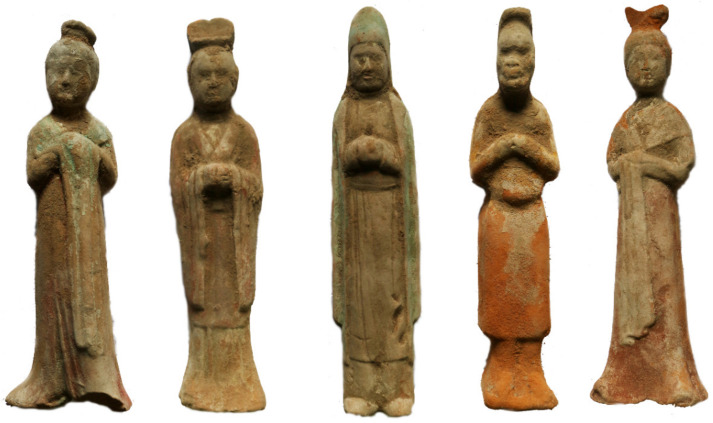
Photograph of the pottery figurines.

More than 400 painted pottery figurines were excavated from this tomb. These painted pottery figurines come in various shapes and are lifelike (Figure 2). The painted female figurines have natural lines and beautiful shapes that fully embody the beauty of women’s flexibility, typical of women in the Tang Dynasty. The painted male figurines also have different styles of dress and represent different identities. Among these pottery figurines, there are also some eunuch figurines, wearing a round-necked, narrow-sleeved, knee-length robe. The upper body of the eunuch figurines leans slightly forward, with a slightly protruding lower abdomen, and a slender figure. These painted pottery figurines are artistic creations based on the lives of the people at the time, reflecting the social conditions and social patterns of that time. They provide important physical information and historical testimony for the study of the politics, culture, costumes, and funeral systems of the Tang Dynasty.

At present, there are no reports on the pigment, adhesives, and manufacturing techniques used in the production of these painted pottery figurines. Scientific analytical techniques were applied in this study to understand the craftsmanship of painted pottery figurines during the Tang Dynasty. Modern analytical techniques, such as optical microscopy (OM), were used for the observation of the figurines’ morphology [3,4,5,6]; energy X-ray fluorescence spectrometry (EDX) identified the element composition of the pigments [7,8,9]; micro-Raman spectroscopy was an important technique for characterizing the constituents of the pigments [10,11,12,13,14,15,16]; pyrolysis–gas chromatography–mass spectrometry (Py-GC/MS) could be employed to determine the composition of the adhesive [17,18,19]; and a dilatometer (DIL) was used to detect the firing temperature of the pottery figurine [20,21,22].

In this work, the OM, EDX, micro-Raman spectroscopy, and Py-GC/MS techniques concentrated on the identification of the pigment and adhesives of the pottery figurines found in Qibi Ming’s tomb. Then, the DIL was used to detect the firing temperature of the pottery figurine. This work provides a more comprehensive understanding of painted pottery figurines from the Tang Dynasty in China.

## 2. Results and Discussion

### 2.1. Surface Morphology of the Pigments

**Figure 3:**
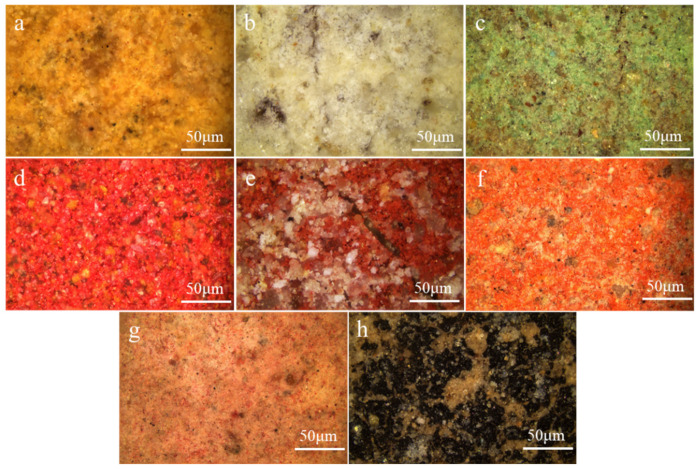
Optical microscopic images of samples (**a**) 10-0852, (**b**) 10-0873, (**c**) 10-0904, (**d**) 10-0675, (**e**) 10-0857, (**f**) 10-0966, (**g**) 10-0678, and (**h**) 10-0888.

To better understand the preservation status of the surface of the pigment layers of the pottery figurines, optical microscopy was carried out to obtain detailed morphology information of the surface of the pigment layers of the samples. Figure 3 shows the pigment layers of the yellow (10-0852), white (10-0873), green (10-0904), red (10-0675, 10-0857, 10-0966), pink (10-0678), and black (10-0888) samples. The pigment layers remained essentially intact. Nevertheless, three types of samples—10-0873, 10-0904, and 10-0857—showed varying degrees of cracking, with the cracking in sample 10-0857 being the most obvious. Sample 10-0888 showed fading, with some pottery substrate being exposed on the outer layer.

### 2.2. Composition of the Pigments

#### 2.2.1. Yellow Pigment Analysis

**Figure 4:**
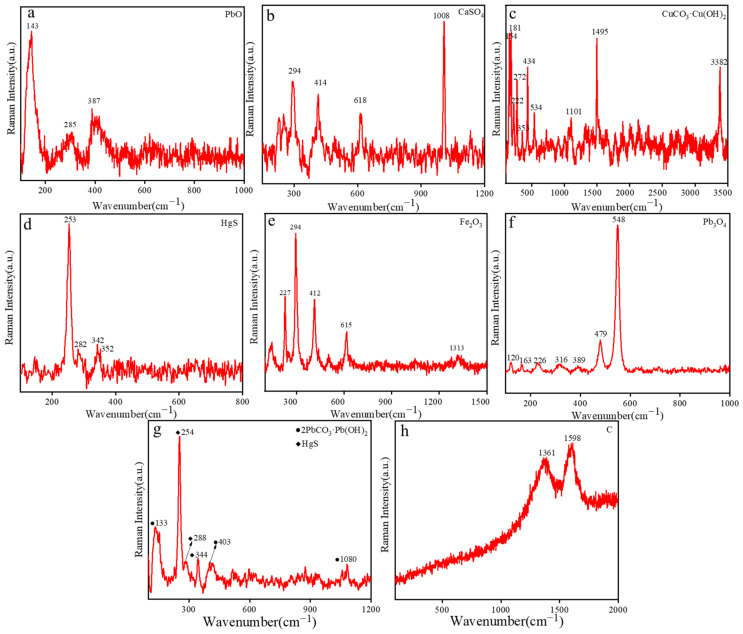
Raman spectra of samples (**a**) 10-0852, (**b**) 10-0873, (**c**) 10-0904, (**d**) 10-0675, (**e**) 10-0857, (**f**) 10-0966, (**g**) 10-0678, and (**h**) 10-0888.

The yellow pigment (10-0852) consisted of Pb, Si, Ca, Fe, K, and Mn, as shown in Table 1. The composition of the yellow pigment was confirmed using micro-Raman spectroscopy. As indicated with Raman peaks at 143 cm^−1^, 285 cm^−1^, and 387 cm^−1^ (Figure 4a), the yellow zones had well-defined and intense peaks centered at 143 cm^−1^, which were attributed to the Pb-O vibration, indicating that the yellow pigment was composed of litharge (PbO) [23]. During the Tang Dynasty, litharge was introduced from Persia. Litharge is originally a transliteration of the Persian “m irdā sang”. However, although the name of litharge comes from a foreign land, it cannot be believed that ancient China did not have such an object. Indeed, in ancient China, the term “Huangdan” was used to refer to “Litharge”. However, before the Sui Dynasty, the name “Huangdan” was shared between both “ Litharge” and “Qiandan”. Later, to avoid confusion, “Litharge” was used specifically to refer to lead monoxide [24].

#### 2.2.2. White Pigment

The white pigment (10-0873) contained Ca, Si, Fe, Pb S, and K. Based on the elemental species, it was determined that the main component of the white pigment was a compound containing Ca. As shown in Figure 4b, the main Raman peaks were recorded at 294 cm^−1^, 414 cm^−1^, 618 cm^−1^, and 1008 cm^−1^. The peak at 1008 cm^−1^ was assigned to the symmetric stretching mode of (SO_4_)^2−^, indicating that the white pigment was gypsum (CaSO_4_). Gypsum has a long history in China. In the Tang Dynasty, gypsum was used as a pigment in construction projects and in various painted cultural relics in China. For example, the famous Da Yan Pagoda and Xiao Yan Pagoda of the Tang Dynasty both used gypsum materials [25,26].

#### 2.2.3. Green Pigment

The green pigment (10-0904) mainly contained elements such as Cu, Si, Ca, Fe, K, S, Pb, and Ti. The main component of the green pigment was a compound containing Cu. As shown in Figure 4c, Raman spectroscopy revealed peaks at 154 cm^−1^, 181 cm^−1^, 222 cm^−1^, 272 cm^−1^, 353 cm^−1^, 434 cm^−1^, 534 cm^−1^, 1101 cm^−1^, 1495 cm^−1^, and 3382 cm^−1^. These are the characteristic Raman peaks of malachite (CuCO_3_·Cu(OH)_2_). The peaks at 154 cm^−1^, 181 cm^−1^, 222 cm^−1^, and 353 cm^−1^ were attributed to the vibration bands of Cu-O. The peak at 1101 cm^−1^ was attributed to (CO_3_)^2−^, and those at 434 cm^−1^, 534 cm^−1^, and 3382 cm^−1^ were the typical Raman peaks of malachite [27]. Malachite has a bright green color, like a peacock’s feather. In ancient China, malachite emerged as the prevailing green mineral pigment, and it was discovered in the tomb of Princess Yongtai of the Tang Dynasty, where malachite was used as the green pigment [28].

#### 2.2.4. Red Pigment

The red pigments included samples 10-0675, 10-0857, and 10-0966. The main elements of sample 10-0675 included Si, Hg, S, Ca, Fe, K, Pb, and Ba. It is plausible to infer that the red pigment (10-0675) might have been cinnabar. As shown in Figure 4d, Raman spectroscopy revealed peaks at 253 cm^−1^, 282 cm^−1^, 342 cm^−1^, and 352 cm^−1^. These are the characteristic Raman peaks of cinnabar (HgS). The peak at 253 cm^−1^ corresponds to the stretching vibration of the Hg-S bonds, while those at 282 cm^−1^ and 342 cm^−1^ were assigned to the degenerate E modes, indicating that the red sample was cinnabar [29]. Cinnabar is a widely used pigment in ancient Chinese paintings, often used in various artistic forms, such as murals, ancient buildings, and grottoes. Its origin can be traced back to the Yangshao culture [30].

The main elements of sample 10-0857 include Fe, Si, Ca, Pb, K, and Ti. Fe is the main element, with the detected high concentration of Fe representing the main composition of hematite (Fe_2_O_3_). To confirm the composition of the pigment, it was necessary to characterize it using micro-Raman spectroscopy. As shown in Figure 4e, the main Raman peaks of the red pigment were observed at 227 cm^−1^, 294 cm^−1^, 412 cm^−1^, 615 cm^−1^, and 1313 cm^−1^. These peaks coincide with the standard spectral peaks of hematite, indicating that the red pigment was indeed hematite (Fe_2_O_3_) [31,32]. Hematite is also one of the main colors that make up painted pottery. Hematite pigments were widely used in ancient China, and many characters wearing red clothes were colored with hematite.

The main elements of sample 10-0857 include Pb, Si, Ca, and K. As shown in Figure 4f, the main Raman peaks of the red pigment were observed at 120 cm^−1^, 163 cm^−1^, 226 cm^−1^, 316 cm^−1^, 389 cm^−1^, 479 cm^−1^, and 548 cm^−1^. These are the characteristic Raman peaks of minium (Pb_3_O_4_). The strong absorption appearing at 548 cm^−1^ was attributed to the stretching of the Pb-O bond. Minium was usually used, besides cinnabar, as a red pigment to achieve red colors. Minium, also known as “red lead” or “true lead”, is a mineral pigment containing lead. Minium has a long history in China and was used as early as the Han Dynasty [29]. The tomb of Murong Zhi, located in the middle branch of the Tang Dynasty’s Silk Road in China, revealed the presence of cinnabar, hematite, and minium [33].

#### 2.2.5. Pink Pigment

The pink pigment (10-0678) contained Pb, Ca, Si, and Hg. Considering the relevant literature, it is plausible to infer that the pink pigment might have been a mixture of white and red pigments [34,35]. As shown in Figure 4g, the main Raman peaks of the pink pigment were observed at 133 cm^−1^, 254 cm^−1^, 288 cm^−1^, 344 cm^−1^, 403 cm^−1^, and 1080 cm^−1^. The peaks at 254 cm^−1^, 288 cm^−1^, and 344 cm^−1^ are the characteristic Raman peaks of cinnabar (HgS) [29].The peaks at 133 cm^−1^, 403 cm^−1^, and 1080 cm^−1^ are the characteristic Raman peaks of white lead (2PbCO_3_·Pb (OH)_2_) [36]. Lead white is the earliest, most widely recorded, and diverse white pigment in Chinese history, with usage records dating back to the Qin Dynasty. The phrase “lead white” comes from the idiom “wash away the lead”. The *Mahamayeri Vidyarajeni Sutra* from the Tang Dynasty unearthed in Lu’an, China, was also tested for lead white [29].

#### 2.2.6. Black Pigment

The black pigment (10-0888) contained Si, Al, Fe, Ca, K, and S. The main Raman peaks, as shown in Figure 4h, were located at 1361 cm^−1^ and 1598 cm^−1^, due to the D-band and G-band of carbon, respectively, indicating that the black pigment corresponded to lead carbon black (C). Carbon black is the earliest nanomaterial used by humans, and China was the earliest country in the world to produce carbon black. As early as over 3000 years ago, Chinese people had mastered the method of burning charcoal to make ink, a method which was already very developed during the Tang Dynasty [3].

### 2.3. Analysis of the Cross-Sections

**Figure 5:**
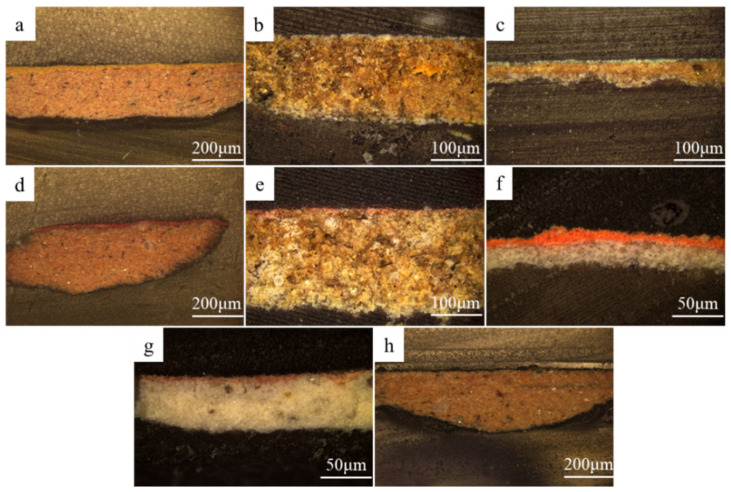
Images of the cross-sections of samples (**a**) 10-0852, (**b**) 10-0873, (**c**) 10-0904, (**d**) 10-0675, (**e**) 10-0857, (**f**) 10-0966, (**g**) 10-0678, and (**h**) 10-0888.

**Figure 6:**
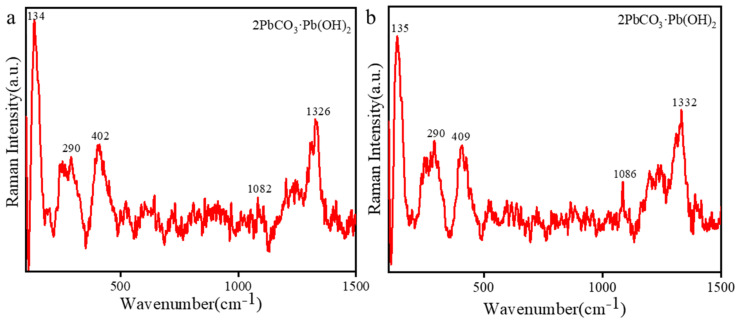
Raman spectra of figurines (**a**) 10-0966 and (**b**) 10-0678.

Cross-sectional analysis helped us understand the production process of the painted pottery figurines [37]. The observation of the cross-sections using OM revealed the painting technique below. The cross-sections of the painted pottery figurines were prepared for observing samples of yellow (10-0852), white (10-0873), green (10-0904), red (10-0675, 10-0857, 10-0966), pink (10-0678), and black (10-0888) pigments, as shown in Figure 5. Two pottery figurines, 10-0966 and 10-0678, had both pigment and preparation layers. These two pottery figurines were made by painting a layer of white pigment on the pottery substrate as a preparation layer and then drawing a thin layer of paint. The other pottery figurines have a pigment layer painted directly onto the pottery substrate, without a preparation layer. The thickness of these painted layers is uneven. As shown in Figure 6a, the main Raman peaks of the red (10-0966) pigment’s preparation layer were observed at 134 cm^−1^, 290 cm^−1^, 402 cm^−1^, 1082 cm^−1^, and 1326 cm^−1^. As shown in Figure 6b, the main Raman peaks of the pink (10-0678) pigment’s preparation layer were observed at 135 cm^−1^, 290 cm^−1^, 409 cm^−1^, 1086 cm^−1^, and 1332 cm^−1^. The preparation layer of the two pottery figurines corresponds to white lead (2PbCO_3_·Pb (OH)_2_) [36].

### 2.4. Analysis of Adhesives

**Figure 7:**
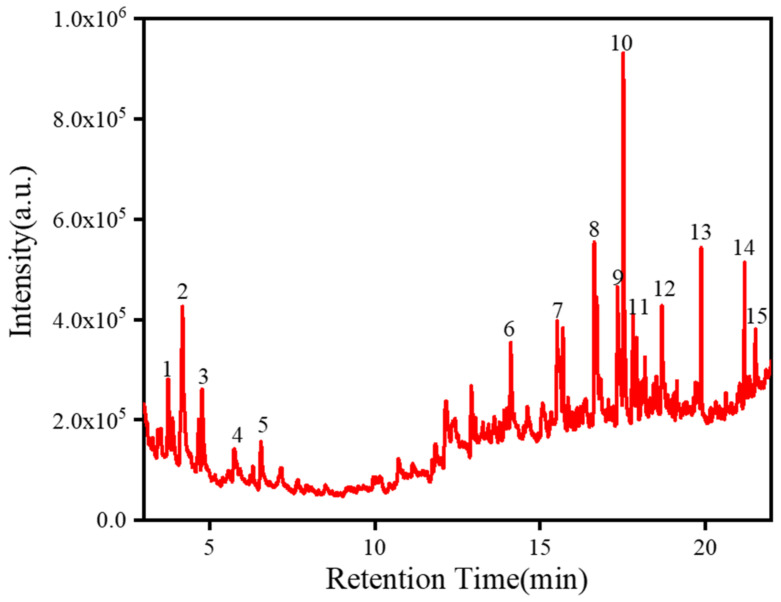
Chromatogram of sample 10-0904.

Adhesives were an important component in our research on the materials and techniques for the production of the painted pottery figurines. Adhesives have always been a hot and difficult issue in the analysis and preservation of cultural heritage. Information about adhesives is usually obtained using Py-GC/MS [10]. The total ion chromatogram of the 10-0904 sample is shown in Figure 7, and the identified compounds with their retention times and peak areas are summarized in Table 2. Pyrrole (peak 1) and toluene (peak 2) compounds were detected in the sample. Pyrrole and its derivatives are the thermal decomposition products of hydroxyproline, while toluene is the thermal decomposition product of phenylalanine. These products serve as the landmark compounds produced after the thermal decomposition of protein adhesives [19,38]. There are research reports according to which only animal glue produces pyrrole and its derivatives after thermal cracking, while other protein adhesives such as egg whites, egg yolks, and pig blood do not contain pyrrole and its derivatives [39]. Due to the fact that only animal glue can be thermally cracked into pyrrole and that no other protein adhesive’s main cracking characteristic compounds had been detected in the sample, it could be determined that the protein adhesive used in the pottery figurines was animal glue. In traditional Chinese painted cultural relics, animal glue, pig blood, and egg whites were commonly used as binders, with animal glue being the most widely used.

Compounds such as isoeugenol (peak 7), veratraldehyde (peak 8), cinnamic acid (peak 11), and menthol (peak 15) were also detected in the sample, indicating that the adhesive also contained aromatic substances such as resveratrol, benzoin, and mint. These compounds not only serve as spices, but also have insecticidal and antibacterial effects [28,40]. Although both palmitic acid (peak 12) and stearic acid (peak 13) were detected in this sample, their content was relatively low and could not be used to determine the presence of vegetable oil in the adhesive [39].

### 2.5. Determining the Firing Temperature of the Pottery Figurines

The principle of measuring the sintering temperature of the pottery figurines using the thermal expansion method was based on the sintering theory assumption found in inorganic materials science, which is that clay undergoes shrinkage sintering during the roasting process. According to this assumption, when a pottery figurine is heated from room temperature to its original firing temperature, it will exhibit a normal, reversible thermal expansion. If the temperature continues to rise, however, irreversible rapid shrinkage will be superimposed on the existing reversible expansion. The starting point of the temperature at which the shrinkage effect begins to superimpose is the basis for determining the figurine’s original firing temperature [41].

**Figure 8:**
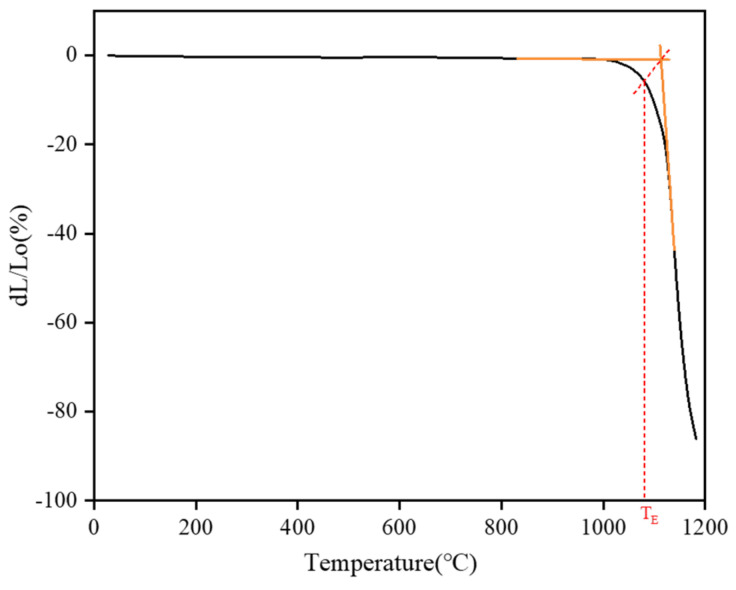
Thermal expansion curve of sample 10-1108.

After the temperature reaches 800 °C, the thermal expansion curve of clay begins to show continuous shrinkage at a certain temperature. Tangents are drawn between the expansion and contraction sections of the curve, and then perpendicular lines are drawn from the intersection of the tangents towards the heating curve. The temperature corresponding to the perpendicular is recorded as T_E_, which is the original firing temperature [20,42]. The results of our experiment are shown in Figure 8. The sample was warmed and terminated at 1185 °C, and the turning point was 1005 °C. The sample continued to shrink after the turning point, and the maximum shrinkage point occurred at 1140 °C. After these calculations, the original firing temperature (T_E_) of the pottery figurines was found to be 1080 °C.

## 3. Materials and Methods

### 3.1. Samples

**Figure 9:**
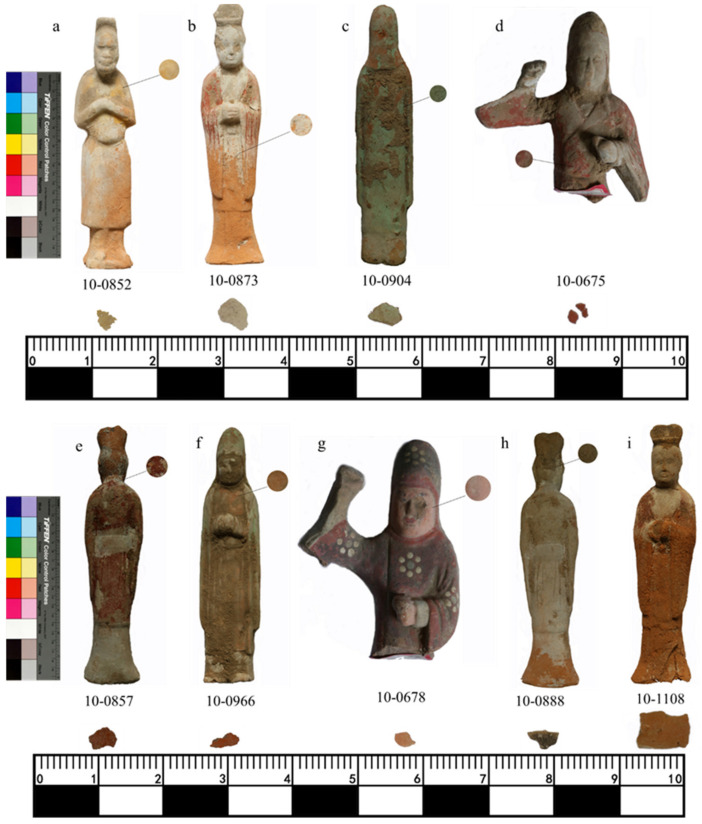
Images of the sampling position of samples (**a**) 10-0852, (**b**) 10-0873, (**c**) 10-0904, (**d**) 10-0675, (**e**) 10-0857, (**f**) 10-0966, (**g**) 10-0678, (**h**) 10-0888, and (**i**) 10-1108.

Sample collection was limited to fragments with small dimensions, and the actual size of each sample collected is shown in Figure 9. The samples mainly comprised yellow (10-0852), white (10-0873), green (10-0904), red (10-0675, 10-0857, 10-0966), pink (10-0678), and black (10-0888) colors and pottery substrate (10-1108). Different analytical methods were used at different stages to obtain comprehensive results; all the analyses in the article were conducted on the collected small fragments.

### 3.2. Cross-Section Sample Preparation

The mold was partially filled with resin (transparent cold mount, Shandong Laizhou Company, Yantai, China) and was allowed to solidify at 25 °C. The sample was placed in the mold, and the remaining space was filled with resin. After curing the upper layer of resin, the sample was extracted using a low-density cutter (DTQ-5, Veiyee, Yantai, China). Sand-paper (600–6000 mesh) was used to polish the surface to achieve a reflective, smooth finish. The structure of the sample’s cross-section was examined with optical microscopy.

### 3.3. Experimental Methods and Instrumentation

The pigments and cross-sections’ characteristics were observed using optical microscopy (OM, BX53M, Olympus, Tokyo, Japan) equipped with 10×, 20×, and 50× objective lens. The microscope was equipped with an achromatic, polarized light, module-collecting lens on a frictionless 360° rotational analyzer with a minimum step of 0.1° and an aperture exceeding 0.90–0.18.

Energy X-ray fluorescence spectrometry (EDX, EDX-7000, Shimadzu, Kyoto, Japan) was employed for the elemental characterization of the pigments. It was equipped with an X-ray tube consisting of an Rh target and a silicon drift detector. This instrument allows for the detection of elements ranging from sodium (Na) to uranium (U). The collected pigment samples were placed on Miramo for testing under atmospheric conditions, and each sample was measured for approximately 5 min.

The micro-Raman spectroscopy was acquired using the Renishaw Invia reflex system (Invia reflex, Renishaw, UK). It was used to analyze the spectral properties of the pigment samples. The spectrum range was from 100 cm^−1^ to 3500 cm^−1^, using a grating with 400 lines/mm and a spot size of 2 μm. The excitation wavelengths were 532 and 785 nm. The objective lens’ magnification was 50×, and the exposure time was 30 s; the accumulation time was 1 s, and the laser power was 2 mW. All spectral treatments were applied using the Wire software v. 3.4 from Renishaw (Wotton-under-Edge, UK).

Pyrolysis–gas chromatography–mass spectrometry (Py-GC/MS) was performed using a combination of a pyrolysis unit (EGA/PY-3030D, Frontier Labs, Koriyama, Japan) and a GC-MS instrument (GC/MS-QP2010Ultra, Shimadzu, Kyoto, Japan). The chromatographic column SLB-5MS (5% diphenyl/95% dimethyl siloxane) was 30 m long, with an internal diameter of 0.25 mm and a film thickness of 0.25 μm (Supelco, Bellefonte, PA, USA). About 2 mg of the sample was taken, powdered, and placed in a thermally cracked sample cup. An aqueous tetramethylammonium hydroxide solution (2 μL) (TMAH, Aladdin, Shanghai, China) with a mass fraction of 25% was added and allowed to settle for 1 h to ensure sufficient contact. Subsequently, it was placed under an infrared lamp and allowed to lyse after the evaporation of water. The pyrolysis temperature, the pyrolysis interface temperature, and the inlet temperature were 600 °C, 300 °C, and 250 °C, respectively. The initial temperature of the chromatographic column was 40 °C. The column was ramped up to 280 °C at a rate of 10 °C/min and maintained for 20 min. The carrier gas for the GC-MS was high-purity helium with an inlet pressure of 15.4 kPa and a splitting ratio of 1:100. The electronic pressure control system was maintained in a constant flow mode. The mass spectrometer was operated using EI ionization at an ionization energy of 70 eV. The scan range of the mass-to-charge ratio was between 50 and 750 (*m*/*z*) with a cycle time of 0.5 s. The isolated chemical compounds were subsequently identified using the NIST14 and corresponding mass spectrometry databases.

The dilatometer (DIL, DIL402, Expedis Classic, Selb, Germany) device is the most accurate device for measuring change in the dimensions of materials caused by thermal expansion. This high degree of temperature sensitivity allows the device to determine the firing temperature of pottery more accurately than other traditional devices [43]. Indeed, this instrument provides the highest level of accuracy, reproducibility, and long-term stability at temperatures of up to 1600 °C. It adopts an Invar measurement system, high-resolution displacement sensors, and comprehensive constant temperature control. In our study, a sample was placed on an Al_2_O_3_ holder and heated at a rate of 5 °C/min in a nitrogen gas atmosphere. The sampling rate was 60 pts min^−1^. Before testing the samples, the system error was adjusted. If the system error is not corrected in advance, the thermal expansion curve will display the rate of change of the sample and the rate of change of the machine’s support system. Errors can be corrected by establishing a baseline thermal expansion curve for standard materials and then comparing the thermal expansion curve of unknown samples with the baseline. Due to the use of a frame and push rod made of aluminum oxide for this device, we chose aluminum oxide as the standard sample. All the samples and the standard samples were placed on brackets, with a thin alumina wafer between the sample and the push rod. This is because aluminum oxide could prevent adhesion between the push rod and the ceramic sample. In the Proteus^®^, the calibration measurement option was selected so that the machine could internally correct errors.

## 4. Conclusions

The pigment, adhesive, and firing temperature employed in the painted pottery figurines from the tomb of Qibi Ming were comprehensively investigated using a range of analytical techniques. The yellow, white, green, red, and black pigments corresponded to litharge (PbO), gypsum (CaSO_4_), malachite (CuCO_3_·Cu(OH)_2_), cinnabar (HgS), hematite (Fe_2_O_3_), minium (Pb_3_O_4_), and carbon black (C), respectively. The pink pigment was a mixture of lead white (2PbCO_3_·Pb (OH)_2_) and cinnabar (HgS), in a particular proportion. The cross-sectional images showed that only two samples, 10-0966 and 10-0678, presented a painted layer that included both a pigment layer and a preparation layer. The preparation layer of both of these pigments was lead white (2PbCO_3_·Pb (OH)_2_). The main component of the adhesive in sample 10-0904 was animal glue, which also contained some aromatic compounds. The firing temperature of the pottery figurines was likely 1080 °C. In summary, this investigation provided scientific support for the archaeologists studying these figurines.

## Figures and Tables

**Table 1:** Elements detected in the pigment particles with EDX.

Sample	Color	Elements
10-0852	Yellow	Pb, Si, Ca, Fe, K, Mn
10-0873	White	Ca, Si, Fe, Pb, S, K
10-0904	Green	Cu, Si, Ca, Fe, K, S, Pb, Ti
10-0675	Red	Si, Hg, S, Ca, Fe, K, Pb, Ba
10-0857		Fe, Si, Ca, Pb, K, Ti
10-0966		Pb, Si, Ca, K
10-0678	Pink	Pb, Ca, Si, Hg
10-0888	Black	Si, Al, Fe, Ca, K, S

**Table 2:** Compounds identified using Py-GC/MS in the total ion chromatogram of sample 10-0904.

Peak Number	Retention Time (min)	Peak Area	Identified Compound
1	3.739	562,115	N-Methyl pyrrole
2	4.174	1,941,906	Toluene
3	4.768	619,003	Alpha-methyl-gamma-butyrolactone
4	5.746	361,143	2-Octyne
5	6.552	561,330	1,7-Octanediyne
6	14.108	512,429	3,4-Dimethoxytoluene
7	15.514	1,048,921	Isoeugenol
8	16.635	2,368,855	Veratraldehyde
9	17.342	1,147,657	2,4-Dimethoxyacetophenone
10	17.514	2,133,439	3,4-Dimethoxybenzoate methyl ester
11	17.812	773,318	2,3-Dimethoxycinnamic acid
12	18.683	808,835	Methyl palmitate
13	19.875	368,136	Methyl stearate
14	21.183	460,770	2,2,5,5-Tetramethoxybiphenyl
15	21.513	263,101	Menthol

## Data Availability

Data are contained within the article.
